# New Advances in Cervical Cancer: From Bench to Bedside

**DOI:** 10.3390/ijerph19127094

**Published:** 2022-06-09

**Authors:** Ottavia D’Oria, Giacomo Corrado, Antonio Simone Laganà, Vito Chiantera, Enrico Vizza, Andrea Giannini

**Affiliations:** 1 PhD Course in “Translational Medicine and Oncology”, Department of Medical and Surgical Sciences and Translational Medicine, Sapienza University, 00185 Rome, Italy; ottavia.doria@uniroma1.it; 2 Dipartimento Scienze della Salute della Donna, del Bambino, e di Sanità Pubblica, Ginecologia Oncologica, Fondazione Policlinico Universitario A. Gemelli IRCCS, 00168 Rome, Italy; giacomo.corrado@policlinicogemelli.it; 3 Unit of Gynecologic Oncology, ARNAS “Civico–Di Cristina–Benfratelli”, Department of Health Promotion, Mother and Child Care, Internal Medicine and Medical Specialties (PROMISE), University of Palermo, 90127 Palermo, Italy; antoniosimone.lagana@unipa.it (A.S.L.); vito.chiantera@gmail.com (V.C.); 4 Gynecologic Oncology Unit, Department of Experimental Clinical Oncology, IRCSS-Regina Elena National Cancer Institute, 00144 Rome, Italy; enrico.vizza@ifo.it

**Keywords:** cervical cancer, cancer therapy, gynecological cancer surgery, targeted therapy

## Abstract

Cervical cancer is the most common gynecologic malignancy and the fourth most common cancer in women worldwide. Over the last two decades, minimally invasive surgery (MIS) has emerged as the mainstay in the surgical management of early-stage cervical cancer, bringing advantages such as a lower operative morbidity and shorter hospital stay compared to open surgery, while maintaining comparable oncologic outcomes in numerous retrospective studies. Considering oncological patients, it is mandatory to assess the oncological outcomes and safety of this type of surgery. Moreover, there are different future outlooks on cervical cancer therapy, based on immunotherapy, target therapy, and poly-ADP-ribose polymerases (PARP) inhibitors in combination with each other, and in combination with standard chemotherapy and radiotherapy. The goal is to find an approach that is as personalized as possible.

## 1. The Current Scenario

Cervical cancer is the fourth most common female cancer worldwide and one of the top three cancers to affect women younger than 45. Despite a decrease in cervical cancer occurrences in developed countries due to the screening program, the frequency of this disease in developing nations continues to increase at an alarming rate caused, at least in part, by insufficient human papillomavirus (HPV) screening and follow-up [1,2,3].

Treatment options for early-stage and locally invasive cervical cancer include radical hysterectomy or radical trachelectomy with pelvic lymphadenectomy and concurrent chemo- and radiotherapy. The treatment options and prognosis for cervical cancer are highly related to disease stage according to International Federation of Gynecology and Obstetrics (FIGO) system [4]. Minimally invasive surgery (MIS) has been proved to be associated with many benefits in the management of different gynecological cancers, in terms of reduced postoperative morbidity, improved recovery and reduced inpatient stay. For oncological patients, it is mandatory to assess the oncological outcomes and safety of this type of surgery. In addition, the role of precancerous lesions treatment is gaining increasing attention. Indeed, the World Health Assembly in 2020 called for the “Elimination of Cervical Cancer” by 2030 by achieving the following three targets: vaccination, screening, and precancerous lesions treatment [5].

## 2. Minimally Invasive Surgery: Still a Safe Option?

The role of MIS (including both laparoscopic and robotic surgery) compared with open surgery for the surgical treatment of early-stage cervical cancer (ECC) is debated. The first randomized control trial, namely, the Laparoscopic Approach to Cervical Cancer (LACC) trial, had unexpectedly reported inferior oncological outcomes with lower survival rates for women who underwent MIS compared with women who underwent open surgery, showing lower disease-free survival (DFS) and overall survival (OS), and creating a large controversy [6]. From that point, different studies were conducted, hoping to deny these findings and re-establish the role of MIS for this purpose. Indeed, MIS has demonstrated survival rates similar to, and fewer complications than, open surgery in several retrospective studies [7]. A recent systematic review and meta-analysis, including 3196 patients, aimed to prove the safety of Laparo-Assisted Vaginal Hysterectomy (LAVRH) in early-stage cervical cancer. LARVH does not affect DFS and OS in ECC patient, and showed overlapping results in the open surgery group [8]. A multicentric Italian study, considering the oncological outcome of patients with FIGO stage IA (positive lymphovascular space invasion)−IB1, tried to investigate the possible surgery-related factors associated with poorer oncologic outcomes in patients who underwent laparoscopic surgery; they concluded that tumor size (≥2 cm) represents the most important risk factor influencing the oncological outcomes [9]. After the LACC trial was closed, two large population-based studies from the Nordic countries (unpublished data) showed no difference in either DFS or OS between MIS and a laparotomic approach [6]. The majority of women in these studies were operated on by robot-assisted surgery. In this scenario, the ongoing robot-assisted approach to cervical cancer (RACC) randomized clinical trial is trying to assess the oncologic safety of this technique for the surgical treatment of ECC as compared with standard laparotomy, in terms of recurrence-free survival (RFS), OS and quality of life [10]. Interestingly, a recent analysis has confirmed that, in women with FIGO stage Ib1-IIa2 cervical adenocarcinoma (CA), there were no significant differences between MIS and open surgery in terms of DFS and OS [11,12]. Nevertheless, further analyses of larger series are needed to better investigate the effect of MIS on the survival rate of ECC patients.

## 3. Future Outlook on Cervical Cancer Therapy

Different clinical trials have been conducted regarding immune checkpoint inhibitors (ICIs) and tumor-infiltrating lymphocytes (TILs) in cervical cancer [13,14]. ICIs act by releasing the so-called immune-suppressing brakes, including programmed death 1 (PD-1), its ligands programmed death ligand 1 (PD-L1) and programmed death ligand 2 (PD-L2), and cytotoxic T-lymphocyte-associated protein 4 (CTLA-4) [15].

Lastly, promising results from adoptive T cell therapy (ACT) include cervical cancer. This approach involves collecting TILs from either the tumor tissue or blood of patients, expanding them ex vivo, and reinfusing them into the patient to effectively target cancer cells, particularly in the LN-145 TIL, an ACT in an ongoing phase-II trial [14]. Moreover, interleukin-2 (IL-2) seems to be a promising option for the treatment of patients with recurrent and/or metastatic cervical cancer. In this regard, different approaches to cervical cancer therapy could be achieved, by combining existing immunotherapies either with other immunotherapies or existing current therapies to obtain better response and survival rates compared with standard treatments. At present, immunotherapies have transformed the management of many solid tumors, including cervical cancer, and this approach is constantly evolving. For instance, in 2018, the American Food and Drug Administration (FDA) approved the programmed death 1 (PD-1) inhibitor pembrolizumab for patients with recurrent or metastatic cervical cancer whose tumors express PD-L1 [16].

Before 2021, pembrolizumab was the only United States Food and Drug Administration-approved immunotherapy in cervical cancer, used specifically for second-line recurrent or metastatic (r/m) disease. Later, tisotumab was approved for second-line r/m cervical cancer, and pembrolizumab combined with chemotherapy ± bevacizumab was approved for first-line r/m disease based on the results of the KEYNOTE-826 study. In 2024, the results of different clinical trials are expected, considering that immunotherapy has the opportunity to establish new standards of care in the treatment of cervical cancer, and new biomarkers can be used to identify the ideal patient populations for these therapies [17].

Targeted therapy is the new accurate therapeutic strategy, using different means of inhibiting different proteins, which are specifically expressed by cancer cells and are responsible for controlling the growth, proliferation and spread of cancer, such as p53, tyrosine kinase Wee1, epidermal growth factor receptor (EGFR), vascular endothelial growth factor (VEGF) [13,14].

An additional strategy to inhibit cervical cancer includes Poly ADP-ribose polymerases (PARP), which are involved in double-strand DNA break repair by homologous recombination; their inhibition was found to enhance the cytotoxicity of DNA-damaging agents, contributing to the development of tumors [18]. Clinical trials have already investigated the PARP-specific targeted therapeutics, veliparib and Olaparib, in patients with advanced, persistent or recurrent cervical cancer [19].

Increasing evidence suggests that cancer stem cells (CSCs) may play an important role in cervical cancer, in terms of relapse, metastasis and chemo/radio-resistance, and of the molecular pathogenesis of this tumor. Several research groups are attempting to identify new target genes, proteins, and signaling pathways that are involved in the stemness of cervical cancer cells. Novel markers for cervical CSCs are being identified and further investigated in the hope of obtaining diverse therapeutic options to cure cervical cancer [20].

## 4. The Combination Therapy in Cervical Cancer

Due to the complexity of cervical cancer, a combination of chemotherapy with either radiotherapy, immunotherapy or targeted therapy has been explored for the management of this type of tumor.

The role of chemotherapy in association with radiotherapy is well-known for the inhibition of micro-metastasis and the reduction in tumor size; combining chemotherapy with immunotherapy may be a promising development in cervical cancer therapy. The combination of standard chemotherapeutic therapies with VEGF antibodies prolongs PFS and OS [20]. In the same way, the combination of bevacizumab with cisplatin and either paclitaxel or topotecan showed an improved median OS compared with chemotherapy alone [21]. In addition, combining therapeutic strategies reduces the toxicity and the adverse effects associated with high doses of monotherapy [22]. However, despite these promising results, further investigation is required of this topic.

## 5. Molecular Markers to Predict Prognosis

Tumor size, stromal invasion, lymphovascular space involvement (LVSI), pathologically confirmed lymph node metastases, extensions into parametrial tissue or positive surgical margins are considered as predictors of recurrence after primary surgery [23].

Advances in biotechnology allowed for the detection of small amounts of RNA and DNA, as well as tumor cells, in a patient’s peripheral blood: circulating tumor cells (CTC), circulating cell-free DNA (cfDNA), circulating HPV DNA and miRNA. Circulating molecules and biomarkers represent a promising diagnostic, prognostic and dynamic tool and can also be easily performed as a liquid biopsy for cervical cancer [24]. Different studies have confirmed the negative impact of CTC detection on PFS in cervical cancer and have confirmed that a more pronounced decrease in CTC count is associated with a lower risk of death [25].

Several groups have investigated singular circulating miRNAs in liquid biopsies: a high level of miR-205 was associated with lymph node metastases, resulting in worse OS. miR-21 detection in cervical cancer patients was also associated with a significantly higher incidence of lymph node metastases, while low levels of miR-218 were associated with a higher tumor volume [26,27].

Nevertheless, to date, protein biomarkers are not routinely used to predict therapy response and survival due, at least in part, to the large number of dysregulated pathways that can overlap. In the future, an accurate prediction of treatment response and survival will help to implement personalized therapies that may improve the treatment of cervical cancer patients.
